# Body Pain and Depressive Symptoms: Patterns and Associations in Middle-Aged and Older Chinese Adults

**DOI:** 10.1155/da/4027080

**Published:** 2025-06-17

**Authors:** Xiaohui Wu, Ruyan Huang, Yue Fei, Rongrong Zhong, Shuo Wang, Xiaojia Huang, Zuowei Wang, Yuncheng Zhu, Yiru Fang

**Affiliations:** ^1^Clinical Research Centre and Division of Mood Disorders, Shanghai Mental Health Center, Shanghai Jiao Tong University School of Medicine, Shanghai 200030, China; ^2^Division of Mood Disorders, Shanghai Hongkou Mental Health Center, Shanghai 200083, China; ^3^Department of Psychiatry and Affective Disorders Center, Ruijin Hospital Affiliated to Shanghai Jiao Tong University School of Medicine, Shanghai 200025, China; ^4^Shanghai Key Laboratory of Psychotic Disorders, Shanghai 201108, China

**Keywords:** body pain, CHARLS, chronic diseases, depression, older adults

## Abstract

**Background:** Depressive symptoms (DS) and body pain are prevalent conditions that significantly impact the quality of life of older adults, often co-occurring with chronic diseases. This study aimed to explore the patterns of body pain characteristics and their association with DS among middle-aged and older Chinese adults.

**Methods:** This cross-sectional study analysed data from 16,039 participants aged ≥45 years in the 2020 wave of the China Health and Retirement Longitudinal Study (CHARLS). DS were assessed using the 10-item Centre for Epidemiologic Studies Depression Scale (CESD-10). Body pain characteristics included pain severity and location. Multiple linear regression and mediation analyses were conducted to examine the relationships between chronic diseases, body pain, and DS.

**Results:** Among participants, 5442 (33.9%) reported DS (CESD-10 score ≥12). The DS group showed significantly higher body pain severity (2.66 ± 1.42 vs. 1.84 ± 1.11, *p* < 0.001) and more painful body parts (5.06 ± 3.87 vs. 3.68 ± 3.03, *p* < 0.001) compared to the non-depressive group. Both pain severity and number of pain sites were independently associated with higher CESD-10 scores. Mediation analysis revealed that body pain severity mediated 29.0% of the total effect between chronic diseases and DS.

**Conclusion:** Body pain plays a significant mediating role in the relationship between chronic diseases and DS among middle-aged and older Chinese adults. These findings emphasize the importance of implementing comprehensive healthcare approaches that integrate pain management with mental health support in primary care settings.

## 1. Introduction

Depression and body pain are two prevalent conditions that significantly impact the quality of life and overall health of individuals, often co-occurring in various populations. Depression represents a significant public health challenge and is one of the main causes of emotional distress among middle-aged and older adults worldwide [1]. According to the World Health Organization, depression is the leading cause of disability globally, with over 280 million people affected, and older adults face a disproportionate burden [2]. Studies have shown that approximately 33%–37% of community-dwelling adults aged 45 years and older in China experience depressive symptoms (DS)[3, 4]. These demographic experiences have higher mortality rates, with depression increasing the risk of all-cause mortality by 1.5–2.0 times [5]. Furthermore, depression in older adults is associated with increased healthcare utilization, reduced quality of life, and annual economic costs exceeding 70 billion CNY in China alone [6]. The burden is particularly severe when depression co-occurs with chronic pain, leading to a substantial increase in functional disability and healthcare utilization [7].

Similarly, body pain, including chronic pain, is a leading cause of disability worldwide, affecting millions of people each year [8]. The global prevalence of chronic pain in adults aged 45 and older ranges from 25% to 50%, with significant variation across regions [9, 10]. In China, recent studies indicate that 28.6% of middle-aged and older adults experience chronic pain, with higher rates in rural areas (32.4%) compared to urban settings (24.8%) [11]. The economic burden of chronic pain in this population is substantial, accounting for 3%–10% of gross domestic product in developed countries through healthcare costs and lost productivity [12]. Recent studies have shown a strong relationship between body pain and DS, suggesting that individuals experiencing chronic pain are at a heightened risk for developing DS [13, 14].

The prevalence of both depression and chronic pain in older adults is of particular concern, as these conditions often exacerbate one another, leading to a downward spiral of deteriorating physical and mental health [15–17]. Previous research had also demonstrated that different dimensions of pain may have distinct associations with DS, with multisite pain, pain severity, and frequency being the strongest predictors of late-life depression [18]. Furthermore, multimorbidity, defined as the presence of two or more chronic conditions, affects nearly 50% of middle-aged and older Chinese adults [19]. The complex interplay between multiple chronic conditions often leads to increased pain severity and more widespread pain distribution, which may significantly impact mental health outcomes [20, 21]. The relationship between chronic conditions, pain, and DS appears to be bidirectional and may operate through various pathways, including sleep disturbances and neurological mechanisms [22]. Research has identified potential pathways through which chronic diseases may lead to depression. First, chronic diseases may directly cause body pain, which serves as a significant risk factor for depression. Second, chronic diseases and pain may share common biological mechanisms with depression [23]. These intricate relationships underscore the profound interconnection among chronic diseases, body pain, and depression, highlighting the critical importance of global medical approaches [24]. Moving forward, comprehensive research and clinical practices must prioritize integrated healthcare strategies that address both physical and psychological well-being of patients with multimorbidity [25].

However, most existing evidence comes from high-income countries or regional studies in China, leaving national patterns largely unexplored. The association of body pain and depression has not been well-established in the Chinese context, and the relationship between overall chronic disease burden and current DS within a recent, large national sample remains less clearly quantified. Therefore, this study utilizes data from the 2020 wave of the China Health and Retirement Longitudinal Study (CHARLS) to fill this gap. We aim to describe the current patterns of body pain characteristics among middle-aged and older Chinese adults, and quantify the distinct mediating roles these pain characteristics play in the association between chronic disease burdens and depressive symptom severity.

## 2. Methods

### 2.1. Study Design and Participants

This study utilized data from the CHARLS, which is a nationally representative longitudinal survey designed to examine the health and economic adjustments to rapidly aging population in China (https://charls.pku.edu.cn/) [26]. CHARLS collects extensive longitudinal data on health, economic, and social factors for adults aged 45 and older, making it an invaluable resource for studying the links between physical and mental health. CHARLS collects comprehensive information on demographics, socioeconomic status, and health conditions through face-to-face computer-assisted personal interviews [27]. The baseline survey was conducted between 2011 and 2012, covering 150 counties/districts in 28 provinces, and 450 villages/communities, with 17,708 participants from 10,257 households being interviewed [28]. To maintain the representativeness of the study population aged 45 and above, CHARLS incorporated a refreshment sample strategy. Additionally, continuous efforts were made in each follow-up wave to trace and interview baseline participants who were not reached in previous waves. Consequently, the total number of participants (including main respondents and their spouses) increased from 17,708 at baseline to 19,395 in the fifth wave of the survey conducted in 2020 [29]. Data collection for the 2020 wave occurred during the COVID-19 pandemic.

The fifth wave of CHARLS in 2020 included a total of 19,395 participants. For this study, we included participants aged 45 years and older who had complete data on basic individual information and the CESD-10 DS assessment. After applying these criteria (complete data on basic info and CESD-10), 3356 participants were excluded due to missing data on these key variables.

### 2.2. Measurement of Depression

DS were assessed using the 10-item Centre for Epidemiological Studies Depression Scale (CESD-10), which has been extensively validated for use in general populations and demonstrates robust reliability and validity for older adults living in community settings in China [30]. It is important to note that the CESD-10 is a screening tool for DS, and the scores indicate symptom levels, not a clinical diagnosis of depression. The CESD-10 consists of questions about the frequency of experiencing the following symptoms during the past week: (1) bothered by things, (2) had trouble keeping mind on what was doing, (3) felt depressed, (4) felt everything was an effort, (5) felt hopeful about future, (6) felt fearful, (7) sleep was restless, (8) was happy, (9) felt lonely, and (10) could not get going. Each item was scored on a 4-point scale: rarely or none of the time (<1 day) = 0, some or a little of the time (1–2 days) = 1, occasionally or moderate amount of time (3–4 days) = 2, and most or all of the time (5–7 days) = 3. The total score ranges from 0 to 30, with higher scores indicating more severe DS. Participants were categorized into two groups: no DS group (NDS group, CESD-10 score <12) and DS group (CESD-10 score ≥12) [31, 32].

### 2.3. Assessment of Body Pain and Chronic Diseases

Body pain was assessed by asking participants whether they were troubled with body pain (none, a little, somewhat, quite a bit, or very severe, scored from 0 to 4) and the specific locations of pain including head, shoulder, arm, wrist, fingers, chest, stomach, back, waist, buttocks, leg, knees, ankle, toes, neck and other parts [33]. The number of painful body parts was computed by summing the total count of these distinct locations. Chronic diseases were determined through self-reported physician diagnoses, including hypertension, dyslipidaemia (high/low blood lipids), hyperglycemia, cancer (excluding minor skin cancers), chronic pulmonary diseases, liver disease (except fatty liver), cardiac disease (including heart attack, coronary heart disease, angina, congestive heart failure, and other heart problems), stroke, kidney disease, digestive system disease, mental health issues, memory-related disease (Alzheimer's disease, brain atrophy), Parkinson's disease, arthritis/rheumatism, and asthma. The number of chronic diseases was calculated as the simple sum of these distinct conditions self-reported by each participant as diagnosed by a physician.

### 2.4. Statistical Analysis

Descriptive statistics were presented as frequencies and percentages for categorical variables, and means ± standard deviations (SDs) for continuous variables. For continuous variables, Student's *t*-test was used for normally distributed data while Mann–Whitney *U* test was applied for non-normally distributed data. Chi-square tests were used for categorical variables.

Cohen's d was calculated as an effect size measure for the *t*-test, with *d* <0.2 considered negligible, 0.2≤ *d* <0.5 small, 0.5≤ *d* <0.8 medium, and *d* ≥0.8 large effect. The effect size for Mann–Whitney *U* tests was calculated using the correlation coefficient *r*, with *r* <0.1 considered negligible, 0.1 ≤ *r* <0.3 small, 0.3≤ *r* <0.5 medium, and *r* ≥0.5 large effect. Effect sizes for chi-square test were calculated using Phi (*φ*) coefficient for 2 × 2 contingency tables and Cramer's *V* for larger contingency tables, with φ/V < 0.1 negligible, 0.1–0.3 small, 0.3–0.5 medium, and ≥0.5 large effect. The relationship between DS severity (measured by CESD-10 score) and body pain (measured by body pain severity and number of painful body parts) was examined using multiple linear regression models, including potential confounders such as age, marital status, number of children, and current status of smoking and drinking. We also performed above multiple linear regression analysis for subgroups by sex. To examine the mediating role of body pain in the relationship between chronic diseases and depression, mediation analysis was conducted using the “mediation” package in R. The analysis followed Baron and Kenny's approach by examining, controlled for covariates of age and sex: (1) the effect of chronic diseases on body pain (path a), (2) the effect of body pain on DS controlling for chronic diseases (path b), and (3) the direct effect of chronic diseases on DS (path c"). The average causal mediation effect (ACME), average direct effect (ADE), and total effect were estimated. The significance of the mediation effect was tested using a nonparametric bootstrap with 1000 resamples to obtain 95% confidence intervals. All statistical analyses were performed using R version 4.0.3 (R Foundation for Statistical Computing, Vienna, Austria), with *p*  < 0.05 considered statistically significant.

## 3. Results

### 3.1. Sample Characteristics

A total of 16,039 participants were included in this analysis, comprising 10,597 individuals in the NDS group (CESD-10 score <12) and 5,442 in the DS group (CESD-10 score ≥12). The mean age of participants was similar between groups (NDS: 61.62 ± 10.09 years vs. DS: 61.84 ± 10.05 years, *p*=0.152). The proportion of men was comparable between groups (46.7% vs. 47.2%, *p*=0.551), as was marital status (living with spouse: 75.4% vs. 74.7%, *p*=0.377). However, significant differences were observed in health behaviors, with the DS group having lower proportions of current smokers (21.5% vs. 28.6%, *p* < 0.001) and regular drinkers (21.1% vs. 30.8%, *p* < 0.001). See Table 1.

### 3.2. Characteristics of Body Pain and Chronic Diseases Conditions

**Figure 1:**
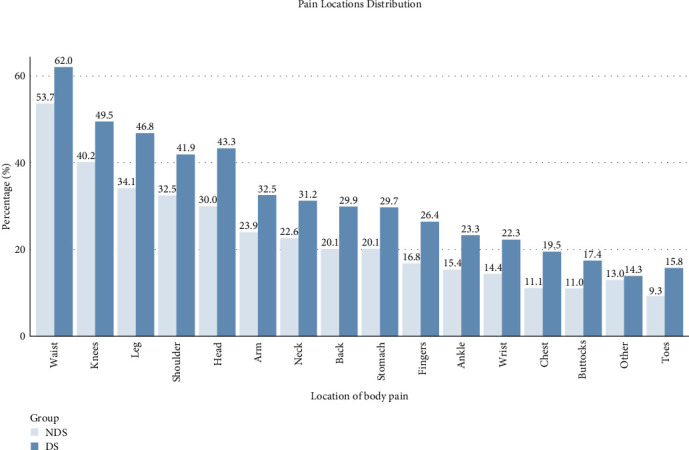
Distribution of body pain locations between NDS and DS groups.

Participants in the DS group reported significantly higher body pain severity (2.66 ± 1.42 vs. 1.84 ± 1.11, *p* < 0.001) and more painful body parts (5.06 ± 3.87 vs. 3.68 ± 3.03, *p* < 0.001) compared with the NDS group. The DS group showed significantly higher prevalence of pain in almost all body locations (Figure 1), particularly in the waist (62.0% vs. 53.7%), knees (49.5% vs. 40.2%), and leg (46.8% vs. 34.1%) (all *p* < 0.001). Among all the body locations, most sites showed negligible to small effects. The strongest effects were observed for head pain (0.138), leg pain (0.129), fingers (0.117), chest (0.117), and back (0.113), all indicating small effects. The DS group also demonstrated a higher burden of chronic diseases with more numbers of chronic diseases (0.73 ± 1.079 vs. 0.47 ± 0.83, *p* < 0.001). Specifically, they showed significantly higher prevalence of hypertension (11.7% vs. 9.6%), dyslipidaemia (12.8% vs. 8.9%), chronic pulmonary diseases (6.3% vs. 3.6%), cardiac diseases (8.1% vs. 5.1%), and arthritis/rheumatism (14.3% vs. 7.9%) (all *p* < 0.001). See Tables 2 and 3. The large effect size for the difference in CESD-10 scores between groups (Cohen's *d* = 2.913) indicates a profound disparity in depressive symptom severity. Similarly, the medium effect size for pain severity (*r* = 0.292) suggests that this difference is not only statistically significant but also clinically meaningful. For body pain locations, while most effect sizes were small (*φ* <0.3), the cumulative impact of multiple pain sites represents a substantial clinical burden, as evidenced by the relationship between pain site count and DS (*β* = 0.37).

### 3.3. Association of Body Pain and DS Severity

**Figure 2:**
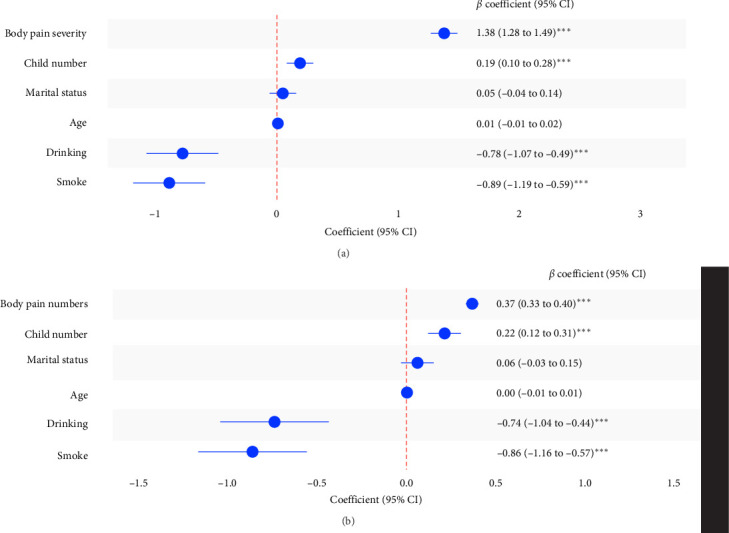
Association between body pain characteristics and depressive symptoms. (A) The association between body pain severity and depressive symptoms. (B) The association between the number of pain sites and depressive symptoms. *⁣*^*∗∗∗*^ Indicates *p* < 0.001.

Multiple linear regression analysis revealed significant associations between body pain and DS after adjusting for age, marital status, number of children, and current status of smoking and drinking. Both pain severity and the number of pain sites were independently associated with higher CESD-10 scores. Body pain severity demonstrated a strong positive association with DS (*β* = 1.38, 95% CI: 1.28–1.49, *p* < 0.001). Similarly, the number of pain sites was also positively associated with depressive symptom severity (*β* = 0.37, 95% CI: 0.33–0.40, *p* < 0.001) (Figure 2). Covariates such as age, marital status, and child number showed weaker or nonsignificant relationships with DS, while smoking and drinking status demonstrated significant negative associations. Similar outcomes were found in both female and male subgroups (Figures S1 and S2).

### 3.4. Mediation Effect of Body Pain Between Chronic Diseases and DS

**Figure 3:**
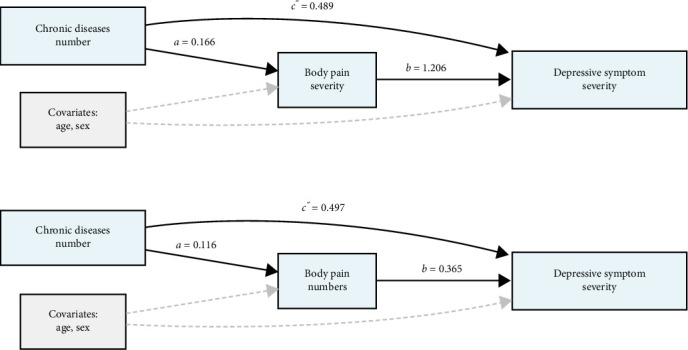
Mediation models of the relationship between chronic diseases numbers and depressive symptom severity through body pain. Path a represents the association between chronic diseases numbers and the mediator, path b represents the association between the mediator and depressive symptom severity, path c” represents the direct effect of chronic diseases numbers on depressive symptom severity.

Mediation analysis revealed that both body pain severity (Model 1) and number of painful sites (Model 2) significantly mediated the relationship between chronic diseases and DS (Figure 3). In Model 1, with body pain severity as the mediator, chronic diseases showed significant direct (*β* = 0.489, 95% CI: 0.350, 0.640, *p* < 0.001) and indirect effects (*β* = 0.200, 95% CI: 0.016, 0.250, *p* < 0.001) on DS. The total effect was 0.689 (95% CI: 0.545, 0.850, *p* < 0.001). Body pain severity mediated 29.0% (95% CI: 22.7%, 38.0%) of the total effect. In Model 2, with body pain sites numbers as the mediator, both direct (*β* = 0.497, 95% CI: 0.349, 0.630, *p* < 0.001) and indirect effects (*β* = 0.149, 95% CI: 0.127, 0.210, *p* < 0.001) remained significant, with a total effect of 0.646 (95% CI: 0.509, 0.810, *p* < 0.001). The proportion of the total effect mediated through body pain numbers was 23.1% (95% CI: 18.6%, 33.0%) (Table S1).

## 4. Discussion

This study explored the association between body pain and DS among middle-aged and older adults in China, utilizing data from the CHARLS. The findings revealed a strong positive relationship between the severity of body pain and the presence of DS, with individuals experiencing more severe or widespread pain exhibiting significantly higher depression scores. Importantly, our mediation analyses demonstrated that both body pain severity and the number of pain sites significantly mediated the relationship between chronic diseases and DS, with body pain severity showing a stronger mediation effect (29.0% of the total effect) compared to the number of pain sites (27.8% of the total effect), highlighting the particular importance of perceived pain intensity in linking physical morbidity to psychological distress. These results emphasize the importance of addressing both physical and mental health challenges concurrently to improve outcomes for aging populations.

Chronic diseases represent a major health challenge for aging populations worldwide, significantly impacting physical function and overall quality of life [34]. The cumulative burden of these conditions not only leads to physical limitations but also serves as a crucial upstream factor contributing to both physical discomfort, such as body pain, and psychological distress, including DS. As evidenced by multiple studies, the presence of chronic conditions like hypertension, diabetes, arthritis, and cardiovascular diseases can severely impact both physical and mental well-being [35, 36]. Body pain, a common consequence of these diseases, exacerbates physical limitations and hinders daily functioning. The elderly population experiences a more profound decline in quality of life due to the interplay of physical discomfort and mental health issues such as depression, resulting in a vicious cycle of worsening health outcomes [37]. Similar to previous studies, pain was identified as a mediating factor between chronic diseases and depression in this study [23]. The social and clinical significance of pain management for the elderly cannot be overstated. Studies show that effective pain management leads to improved quality of life, reduced disability, and a decrease in the psychological burden of chronic pain [38]. Clinically, managing pain in older adults is crucial not only for reducing physical suffering but also for mitigating the risk of depression and improving mental health outcomes. Early intervention in managing pain is vital in preventing the escalation of both physical and mental health conditions. As research has shown, addressing pain early on can help prevent the development of depression, particularly in individuals with chronic illnesses [39]. Early diagnosis of depression, coupled with pain management strategies, is essential for breaking the cycle of suffering. The timely treatment of pain and mental health conditions can improve the life satisfaction and independence of older adults, which is critical in maintaining their overall well-being [40].

Body pain and DS represent a significant comorbid condition with complex bidirectional interactions, as extensively documented in contemporary medical research [41]. Emerging evidence highlights several shared biological mechanisms underlying this comorbidity. Genetic studies have demonstrated that pain phenotypes and depression share overlapping genetic risk factors, with significant positive genetic correlations observed across various types of pain and DS [42]. This suggests that individuals predisposed to one condition may also have a higher vulnerability to the other, potentially due to shared neurobiological pathways. Preclinical research has provided valuable insights into the potential neurobiological mechanisms connecting chronic pain and depression. Animal model studies focusing on both pain and depression have identified brain neuronal inflammatory mediators, including brain interleukin-1 and complement components, as potential key contributors to this relationship [43, 44]. Epigenetic factors, including microRNAs, also play a critical role in central sensitization, which is a hallmark of chronic pain and depression [45]. Chronic pain and depression share complex biological mechanisms involving genetic predispositions, inflammatory processesand epigenetic modulation. These shared pathways underscore the importance of integrative approaches to diagnosis and treatment, targeting common underlying processes rather than addressing each condition in isolation.

The demonstrated mediating role of body pain in the relationship between chronic disease burden and DS underscores the importance of adopting integrated healthcare approaches for middle-aged and older adults. Evidence suggests that care models emphasizing person-centeredness, coordinated multidisciplinary teams, and comprehensive assessments are beneficial for older adults with comorbid physical and mental health conditions [38, 46]. Effective pain assessment and management are crucial, as addressing pain may mitigate the risk or severity of concurrent DS [39]. Current guidelines recommend routine screening for depression in the adult population, including older adults [47], and geriatric assessment protocols often incorporate screening for both pain and depression, particularly in primary care settings where these conditions frequently co-occur [48]. Therefore, implementing comprehensive management plans that integrate evidence-based pharmacological treatments with nonpharmacological strategies for pain alongside appropriate mental health support is essential for improving patient outcomes and addressing the interconnected challenges of chronic disease, pain, and depression in this population. Our findings also suggest that the significant mediating role of pain highlights the potential value of implementing enhanced, bidirectional screening protocols, where individuals reporting significant pain are assessed for depression, and vice versa.

Our study has several important limitations that should be considered when interpreting the results. First, due to the cross-sectional nature of our analysis using the CHARLS 2020 wave data, we cannot establish causal relationships between body pain, chronic diseases, and DS. The bidirectional nature of these relationships requires longitudinal studies to fully elucidate their temporal sequence and causal pathways. Second, both pain and chronic disease assessments relied on self-reported measures, which may be subject to recall bias and potential underreporting, particularly among older adults who might normalize their pain experiences or have cognitive limitations affecting their recall accuracy. Third, while the CESD-10 is a validated screening tool for DS, it cannot replace clinical diagnosis of depression, and the results should be interpreted as indicators of depressive symptomatology rather than clinical depression. Fourth, lack of information on pain duration and pain medication usage could influence the accuracy of the results. Finally, we did not explore the potentially distinct roles of specific types of chronic conditions or specific patterns of comorbidity, which should be an important theme for future research.

## 5. Conclusion

In conclusion, this study highlights the complex interrelationship between body pain, chronic diseases, and DS among middle-aged and older Chinese adults. The significant mediating role of pain in the relationship between chronic diseases and depression underscores the importance of implementing comprehensive healthcare approaches that address both physical and mental health needs. Healthcare providers should consider incorporating routine pain and depression screenings into primary care settings, particularly for older adults with chronic conditions. Additionally, developing integrated care models that combine pain management with mental health support could potentially improve overall health outcomes and quality of life for this vulnerable population. Future research should focus on developing and evaluating targeted interventions that address both pain and depression simultaneously, as well as investigating the long-term impacts of integrated care approaches through longitudinal studies. Such efforts will be crucial in addressing the growing healthcare needs of aging populations and reducing the burden of these interrelated health conditions in China's rapidly aging society.

## Figures and Tables

**Table 1:** Demographic information of selected subjects grouped by CESD-10 score.

Variables	NDS	DS	*χ^2^/t/Z*	*p*	Effect size
*N*	10,597	5442	—	—	—
Age	61.62 ± 10.09	61.84 ± 10.05	1.432	0.152	0.011
Men	4946 (46.7)	2567 (47.2)	0.356	0.551	0.005
Marital status	—	—	0.779	0.377	0.007
Living without spouse	2612 (24.6)	1367 (25.3)	—	—	—
Living with spouse	7985 (75.4)	4066 (74.7)	—	—	—
Number of child	2.46 ± 1.27	2.67 ± 1.37	10.019	<0.001	0.080
Smoking status	—	—	154.400	<0.001	0.076
Still smoke	3028 (28.6)	1169 (21.5)	—	—	—
Quit	1501 (14.2)	607 (11.2)	—	—	—
Never smoked	6068 (57.3)	3666 (67.4)	—	—	—
Drinking status	—	—	186.430	<0.001	0.103
More than once a month	3268 (30.8)	1149 (21.1)	—	—	—
Less than once a month	1080 (10.1)	521 (9.6)	—	—	—
None	6249 (66.1)	3772 (69.3)	—	—	—
CESD-10 score	6.43 ± 2.89	16.60 ± 4.01	104.065	<0.001	2.913

*Note:* NDS, no depressive symptoms group defined by CESD-10 score <12; DS, depressive symptoms group defined by CESD-10 score^3^ 12; CESD-10, the 10-item Centre for Epidemiological Studies Depression Scale. Variables are presented as mean ± SD, or *n* (%). The effect size was calculated as Cohen's *d* for *t*-test, correlation coefficient *r* for Mann–Whitney *U* tests, and Phi (*φ*) coefficient or Cramer's *V* for chi-square test.

**Table 2:** Characteristics of body pain between the NDS and DS groups.

Variables	NDS	DS	*χ* ^2^ */t/Z*	*p*	Effect size
Body pain severity	1.84 ± 1.11	2.66 ± 1.42	37.012	<0.001	0.292
Body parts feeling pain					
Head	1570 (30.0)	1754 (43.3)	176.824	<0.001	0.138
Shoulder	1701 (32.5)	1697 (41.9)	87.538	<0.001	0.097
Arm	1254 (23.9)	1317 (32.5)	83.993	<0.001	0.095
Wrist	754 (14.4)	902 (22.3)	96.756	<0.001	0.102
Fingers	878 (16.8)	1069 (26.4)	127.960	<0.001	0.117
Chest	582 (11.1)	790 (19.5)	127.890	<0.001	0.117
Stomach	1055 (20.1)	1203 (29.7)	113.542	<0.001	0.11
Back	1054 (20.1)	1211 (29.9)	118.495	<0.001	0.113
Waist	2810 (53.7)	2512 (62.0)	65.597	<0.001	0.084
Buttocks	578 (11.0)	705 (17.4)	77.933	<0.001	0.091
Leg	1786 (34.1)	1895 (46.8)	153.887	<0.001	0.129
Knees	2103 (40.2)	2004 (49.5)	80.708	<0.001	0.093
Ancle	804 (15.4)	944 (23.3)	94.736	<0.001	0.101
Toes	487 (9.3)	638 (15.8)	89.439	<0.001	0.098
Neck	1184 (22.6)	1265 (31.2)	87.664	<0.001	0.097
Other	679 (13.0)	578 (14.3)	3.347	0.067	0.019
Numbers of painful parts	3.68 ± 3.03	5.06 ± 3.87	17.691	<0.001	0.184

*Note:* Variables are presented as mean ± SD, or *n* (%). DS, depressive symptoms group; NDS, no depressive symptoms group.

**Table 3:** Chronic diseases conditions between the NDS and DS groups.

Variables	NDS	DS	*χ* ^2^ */t/Z*	*p*	Effect size
Chronic diseases					
Hypertension	714 (9.6)	418 (11.7)	11.219	0.001	0.031
Dyslipidaemia	781 (8.9)	548 (12.8)	49.310	<0.001	0.061
Hyperglycemia	421 (4.4)	302 (6.3)	2.622	<0.001	0.041
Cancer	79 (0.8)	65 (1.2)	8.235	0.004	0.022
Chronic pulmonary diseases	348 (3.6)	298 (6.3)	54.232	<0.001	0.061
Liver diseases	193 (1.9)	159 (3.1)	22.583	<0.001	0.038
Cardiac diseases	471 (5.1)	350 (8.1)	45.912	<0.001	0.058
Stroke	144 (1.4)	157 (3.1)	48.838	<0.001	0.056
Kidney diseases	277 (2.8)	245 (5.0)	47.931	<0.001	0.056
Digestive system diseases	518 (6.3)	368 (10.3)	59.399	<0.001	0.071
Mental health	52 (0.5)	112 (2.1)	89.435	<0.001	0.075
Memory-related diseases	226 (21.)	299 (5.5)	128.439	<0.001	0.089
Parkinson's disease	45 (0.4)	67 (1.2)	33.877	<0.001	0.045
Arthritis rheumatism	615 (7.9)	452 (14.3)	104.504	<0.001	0.098
Asthma	145 (1.4)	136 (2.7)	29.149	<0.001	0.043
Chronic diseases numbers	0.47 ± 0.83	0.73 ± 1.079	15.189	<0.001	0.120

*Note:* Variables are presented as mean ± SD, or *n* (%). DS, depressive symptoms group; NDS, no depressive symptoms group.

## Data Availability

The data that support the findings of this study are available from the corresponding author upon reasonable request.

## Supporting Information

Supporting Information This supporting information provides additional information and data related to the study presented in the main manuscript. It includes detailed analyses of body pain characteristics and depressive symptoms in male and female participants, and comprehensive mediation analysis of the relationship between chronic diseases, body pain, and depressive symptoms. This supporting information is essential for providing further evidence to support the findings presented in the main manuscript. It offers in-depth subgroup analyses and statistical mediation models that reinforce the conclusions about the role of body pain in mediating the relationship between chronic diseases and depressive symptoms. Figure S1: Association between body pain characteristics and depressive symptoms in male participants, including separate analyses of pain severity and pain site count. Figure S2: Association between body pain characteristics and depressive symptoms in female participants, including similar breakdown as Figure S1. Table S1: Mediation analysis illustrating the association between chronic disease numbers and depressive symptom severity, using body pain severity and pain site count as mediators.
