# Supplementary material for: Body Pain and Depressive Symptoms: Patterns and Associations in Middle-Aged and Older Chinese Adults
Source: Depress Anxiety. 2025 Jun 17;2025:4027080. doi: 10.1155/da/4027080 (PMC12187443; doi:10.1155/da/4027080)
Supplement: Supporting Information — This supporting information provides additional information and data related to the study presented in the main manuscript. It includes detailed analyses of body pain characteristics and depressive symptoms in male and female participants, and comprehensive mediation analysis of the relationship between chronic diseases, body pain, and depressive symptoms. This supporting information is essential for providing further evidence to support the findings presented in the main manuscript. It offers in-depth subgroup analyses and statistical mediation models that reinforce the conclusions about the role of body pain in mediating the relationship between chronic diseases and depressive symptoms. Figure S1: Association between body pain characteristics and depressive symptoms in male participants, including separate analyses of pain severity and pain site count. Figure S2: Association between body pain characteristics and depressive symptoms in female participants, including similar breakdown as Figure S1. Table S1: Mediation analysis illustrating the association between chronic disease numbers and depressive symptom severity, using body pain severity and pain site count as mediators. [file 4027080.f1.docx]

**Supplementary Materials**

**Supplementary figures**


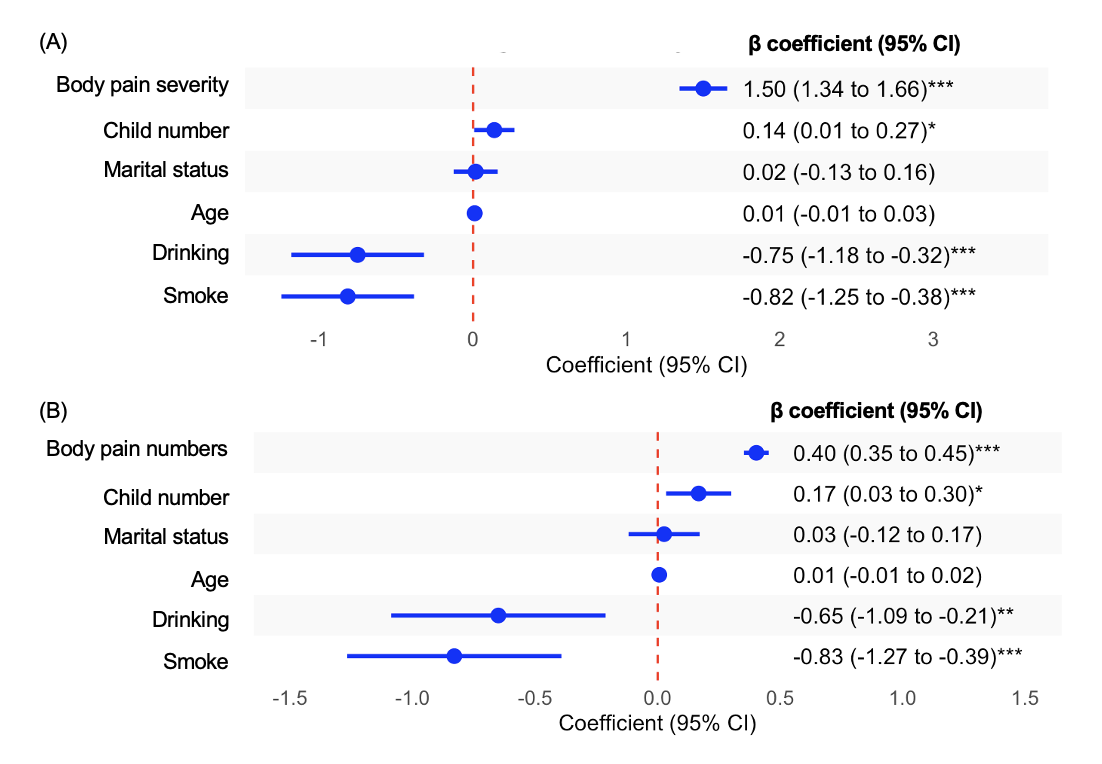


FIGURE S1: Association between body pain characteristics and depressive symptoms in male participants. (A) The association between body pain severity and depressive symptoms. (B) The association between the number of pain sites and depressive symptoms. * Indicates *p* < 0.05, ** indicates *p* < 0.01, *** indicates *p* < 0.001.


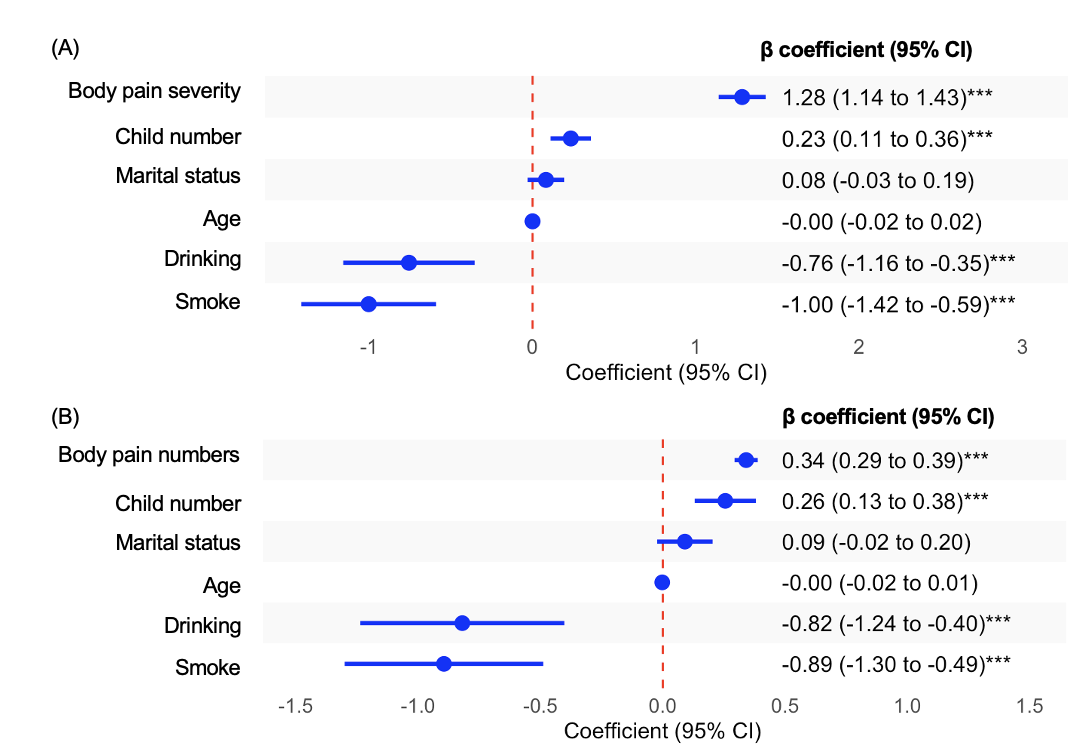


FIGURE S2: Association between body pain characteristics and depressive symptoms in female participants. (A) The association between body pain severity and depressive symptoms. (B) The association between the number of pain sites and depressive symptoms. *** Indicates *p* < 0.001.

**Supplementary tables**

| TABLE S1 : Mediation analysis of the association between chronic diseases numbers and depressive severity through body pain | | | | |  |
| --- | --- | --- | --- | --- | --- |
| Parameter | Model 1 | | Model 2 | |  |
|  | Effect (95% CI) | *p* | Effect (95% CI) | *p* |  |
| ACME | 0.200 (0.160, 0.250) | <0.001 | 0.149 (0.127, 0.210) | <0.001 |  |
| ADE | 0.489 (0.350, 0.640) | <0.001 | 0.497 (0.349, 0.630) | <0.001 |  |
| Total effect | 0.689 (0.545, 0.850) | <0.001 | 0.646 (0.509, 0.810) | <0.001 |  |
| Proportion mediated (%) | 29.0 (22.7, 38.0) | <0.001 | 23.1 (18.6, 33.0) | <0.001 |  |
| Note: The mediation analysis controlled for age and sex.  CI, Confidence Interval; ACME, Average Causal Mediation Effect; ADE, Average Direct Effect. Model 1 used body pain severity as the mediator; Model 2 used body pain numbers as the mediator. All effects were estimated using 1,000 bootstrap resamples. | | | | |  |
|  |  |  |  |  |  |
|  |  |  |  |  |  |
